# Neck-to-height ratio is positively associated with diabetic kidney disease in Chinese patients with type 2 diabetes mellitus

**DOI:** 10.3389/fendo.2022.1100354

**Published:** 2023-01-10

**Authors:** Zhi-Ying He, Xiao Gu, Lin-Jia Du, Xiang Hu, Xing-Xing Zhang, Li-Juan Yang, Ying-Qian Li, Jing Li, Lin-Yu Pan, Bo Yang, Xue-Jiang Gu, Xiu-Li Lin

**Affiliations:** ^1^ Department of Endocrine and Metabolic Disease, The First Affiliated Hospital of Wenzhou Medical University, Wenzhou, China; ^2^ Department of Preventive Medicine, School of Public Health and Management, Wenzhou Medical University, Wenzhou, China; ^3^ Institute of Lipids Medicine, Wenzhou Medical University, Wenzhou, China; ^4^ Department of Infection, The First Affiliated Hospital of Wenzhou Medical University, Wenzhou, China

**Keywords:** upper-body subcutaneous fat, neck-to-height ratio, neck circumference, diabetic kidney disease, interactive analysis

## Abstract

**Introduction:**

The aim of this study was to investigate the associations of neck circumference (NC) and neck-to-height (NHR) with diabetic kidney disease (DKD) in Chinese patients with type 2 diabetes mellitus (T2DM).

**Materials and methods:**

A total of 2,615 patients with prevalent T2DM were enrolled. NHR was calculated through NC (cm) divided by height (cm), and prevalent DKD was defined as the urinary albumin-to-creatinine ratio (UACR) ≥ 30 mg/g or the estimated glomerular filtration rate (eGFR) < 60 ml/min per 1.73 m^2^ in the absence of other primary kidney diseases.

**Results:**

The levels of NC and NHR were higher in DKD patients compared with non-DKD patients (38.22 vs. 37.71, P = 0.003; 0.232 vs. 0.227, P < 0.001, respectively). After full adjustments, individuals at the highest tertile of NHR had higher odds of DKD than those at the lowest tertile (multivariate-adjusted OR = 1.63, 95% CI: 1.22, 2.18), but this association was not pronounced with NC (multivariate-adjusted OR = 1.24, 95% CI: 0.87, 1.76). Individuals at the highest tertile of NHR had lower eGFR (β = -4.64, 95% CI: -6.55, -2.74) and higher UACR levels (β = 0.27, 95% CI: 0.10, 0.45) than those at the lowest tertile. The adverse association between NHR and prevalent DKD remained statistically significant among most of the subgroups analyzed and no interaction effects were observed.

**Conclusion:**

The increase in NHR was adversely and independently associated with DKD in this Chinese T2DM population.

## 1 Introduction

Diabetic kidney disease (DKD), as one of the most common chronic complications of diabetes, is developed in about 20 - 40% of patients with diabetes (1). Patients with DKD are more likely to progress to end-stage renal disease (ESRD), as well as have a higher risk of cardiovascular diseases (CVD) and all-cause mortality (2, 3). It is vital to discover potential markers to identify patients at a higher risk of DKD.

Obesity is proved to be an important risk factor for kidney damage. Adipose tissue releases a mass of signaling molecules, including inflammatory and hormonal factors, which are critical for inter-organ crosstalk. The communication between adipocytes and the kidney, known as the adipo-renal axis is critical for normal kidney function and the effective response of the kidney to injury (4, 5). Meanwhile, plenty of anthropometric indices of obesity, such as body mass index (BMI), waist circumference (WC), waist-to-hip ratio (WHR), and the Chinese visceral adiposity index (CVAI) have already been reported to be related to DKD (6–8).

Upper-body subcutaneous fat, a unique fat depot independent of generalized and central adiposity, could present extra risk for metabolic disorders (9, 10). Evidence to date have suggested that upper-body subcutaneous fat could always be estimated by neck circumference (NC) (11), which as a simple anthropometric index is not affected by clothing or feeding. However, NC as a regional obesity indicator, could not take the overall body fat distribution fully into account. Neck-to-height ratio (NHR), adjusted for the discrepancies in NC attributable to different heights, shows its advantage in reflecting the whole body fat distribution based on height. And accumulating evidence from clinical studies supported that NHR was a better index for the assessment of upper-body subcutaneous fat than NC in patients with metabolic disorders (12–14). Of note, population-based studies that focused on the relationship between upper-body subcutaneous fat and kidney damage are limited. In populations without diabetes, clinical findings suggested that NC was associated with indicators of kidney dysfunction (15–17). Only a Chinese study targeted subjects with diabetes showed that NC was positively associated with the prevalence of DKD (8). Furthermore, there has been no population-based studies to investigate the association between NHR and renal damage.

Therefore, the goal of our study was to explore the associations of NC and NHR with DKD in patients with type 2 diabetes mellitus (T2DM).

## 2 Materials and methods

### 2.1 Study subjects

**Figure 1:**
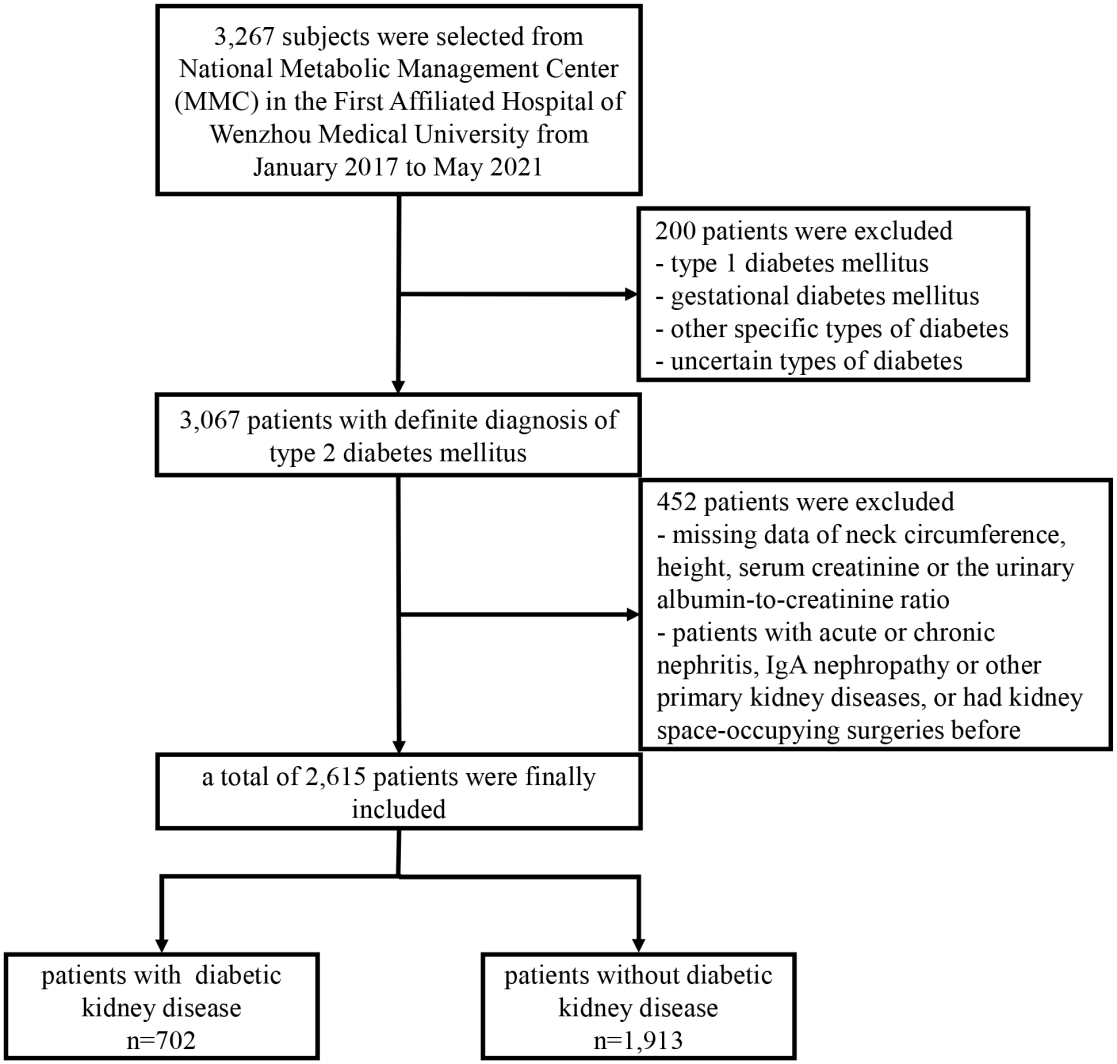
Flowchart of study participants included.

A total of 3267 adults were enrolled from National Metabolic Management Center (MMC) (18, 19) in the First Affiliated Hospital of Wenzhou Medical University from January 2017 to May 2021. 3067 subjects were diagnosed with T2DM according to the diagnostic criteria of World Health Organization (WHO) (20). The exclusion criteria were as followed: 1) patients without data of NC, height measurements, serum creatinine or the urinary albumin–to–creatinine ratio (UACR); 2) patients with acute or chronic nephritis, IgA nephropathy, or other primary kidney diseases, or had kidney space-occupying surgeries before. Finally, 2615 patients were included in the present study. An overview of the patients selected was presented in **Figure 1**.

The study was approved by the Ethics Committee in Clinical Research of the First Affiliated Hospital of Wenzhou Medical University (No: KY2021-173), and all participants have been given written informed consent.

### 2.2 Data collection

Including systolic blood pressure (SBP), diastolic blood pressure (DBP), weight, height, WC and NC were measured by trained staff according to standard protocols. Body weight and standing height were measured accurate to the 0.1 kg and 0.1 cm without shoes or heavy clothes. WC was measured at the midpoint between the lowest rib and the iliac crest. NC was measured with the upper border of a flexible tape placed below the laryngeal prominence and circled vertically to the long axis of the neck (9). BMI was calculated through body weight (kg) divided by the square of height (m^2^). NHR was calculated through NC (cm) divided by height (cm).

Biochemical indicators, including fasting plasma glucose (FBG), glycosylated hemoglobin A1c (HbA1c), triglyceride (TG), total cholesterol (TC), high-density lipoprotein cholesterol (HDL-C), low-density lipoprotein cholesterol (LDL-C), and uric acid (UA) were assayed through venous blood samples obtained in the morning after an overnight fast (≥ 8h). Non-HDL-c was calculated through TC minus HDL-C. Serum creatinine (Cr), urinary albumin and urinary creatinine were measured with an automatic biochemical analyzer (Beckmann AU 5800). UACR was ratios of urinary albumin to urinary creatinine, the estimated glomerular filtration rate (eGFR) was calculated according to the Chronic Kidney Disease Epidemiology Collaboration (CKD-EPI) equation (21).

Diabetes duration, lifestyle factors including education attainment, current smoking and drinking and medication history were all obtained by standardized questionnaires.

### 2.3 Definition of variables

DKD was defined as UACR ≥ 30 mg/g or eGFR < 60 ml/min per 1.73 m^2^, meanwhile in the absence of other primary kidney diseases as suggested by the ADA recommendations (1). Overweight/general obesity was defined as BMI ≥ 24 kg/m^2^, and central obesity was defined as WC ≥ 90 cm for men, ≥ 85 cm for women, all according to the Guideline for the Prevention and Treatment of Type 2 Diabetes Mellitus in China (22). Hypertension was defined as SBP ≥ 140 mmHg and/or DBP ≥ 90 mmHg, or undergoing antihypertensive medication currently (23). Dyslipidemia was defined as TG ≥ 2.3 mmol/L, or TC ≥ 6.2 mmol/L, or HDL-c < 1.0 mmol/L, or LDL-c ≥ 4.1 mmol/L, or non-HDL-c ≥ 4.9 mmol/L, suggested by the 2016 Chinese Guidelines for the Management of Dyslipidemia in Adults (24).

### 2.4 Statistical analysis

All statistical analyses were performed by SPSS version 26.0 software (IBM Corporation). Data were displayed as means ± standard deviation or as median (interquartile range) for continuous variables, numbers and percentage for categorical variables. Discrepancies between subjects with and without DKD were analyzed using Student’s t test for normally distributed continuous variables, Mann-Whitney U test for abnormally distributed continuous variables, and chi-square test for categorical variables. Multivariable logistic regression models were applied to investigate the relationship between DKD and the tertiles of NC and NHR, odds ratios (ORs) and 95% confidence intervals (CIs) were provided. In addition, multivariable linear regression models were used for eGFR and log-transformed UACR (LnUACR) in relation to the tertiles of NHR. Subgroup analyses were conducted to test the potential interactions between NHR and the other cardiometabolic factors on DKD. For regression models that mentioned above: age and sex were adjusted for in model 1; age, sex, diabetes duration, smoking and drinking status, SBP, TC, FBG were adjusted for in model 2; age, sex, diabetes duration, smoking and drinking status, SBP, TC, FBG, BMI, WC, antidiabetic agents currently and antihypertensive agents currently were adjusted for in model 3. All P values were two-sided and considered statistically significant when < 0.05.

## 3 Results

### 3.1 Baseline characteristics

There were 702 (26.85%) patients with DKD and 1913 (73.15%) patients without DKD enrolled in the present study. NC and NHR levels were significantly higher in patients with DKD compared to those without (38.22 vs. 37.71, *P* = 0.003; 0.232 vs. 0.227, *P* < 0.001). In contrast to patients without DKD, those with DKD were older, less educated, and had longer diabetes duration, higher proportions of women and non-smokers, as well as had higher levels of SBP, DBP, BMI, WC, FBG, TG, TC, Cr, UACR, UA, current antidiabetic agents, current antihypertensive agents usage and lower height, eGFR (all *P* < 0.05). There were no statistical differences in current drinking status, HbA1c, HDL-c or LDL-c between the two groups (**Table 1**).

**Table 1:** Baseline characteristics of study participants based on DKD status.

	Total	DKD+	DKD-	P Value
Participants (n, %)	2615	702, 26.85	1913, 73.15	/
Socio-demographics factors				
Age (years)	50.96 ± 10.95	53.29 ± 10.43	50.10 ± 11.02	<0.001
Female (n, %)	829, 31.70	267, 38.03	562, 29.38	<0.001
Education attainment: high school or above (n, %)	633, 24.61	116, 16.89	517, 27.43	<0.001
Diabetes duration (month)	68.0 (12.0, 135.0)	109.5 (41.0, 165.0)	60.0 (4.0, 126.0)	<0.001
Lifestyle risk factors				
Current smoking (n, %)	874, 33.44	202, 28.77	672, 35.15	0.002
Current drinking (n, %)	1142, 43.67	297, 42.31	845, 44.17	0.394
Anthropometric parameters				
SBP (mmHg)	127.26 ± 18.72	134.78 ± 20.77	124.51 ± 17.11	<0.001
DBP (mmHg)	75.42 ± 11.00	77.95 ± 12.00	74.49 ± 10.46	<0.001
BMI (kg/m^2^)	24.67 ± 3.49	25.28 ± 3.71	24.45 ± 3.38	<0.001
WC (cm)	88.98 ± 9.59	90.39 ± 9.97	88.46 ± 9.39	<0.001
NC (cm)	37.85 ± 3.78	38.22 ± 4.07	37.71 ± 3.66	0.003
Height (cm)	164.7 ± 8.4	163.2 ± 8.5	165.3 ± 8.3	<0.001
NHR	0.228 (0.217, 0.241)	0.232 (0.221, 0.245)	0.227 (0.215, 0.239)	<0.001
Biochemical indexes				
FBG (mmol/L)	7.7 (6.1, 9.7)	8.1 (6.2, 10.4)	7.5 (6.0, 9.5)	<0.001
HbA1c (%)	10.09 ± 2.41	10.12 ± 2.35	10.08 ± 2.43	0.660
TG (mmol/L)	1.55 (1.07, 2.35)	1.81 (1.24, 2.70)	1.45 (1.02, 2.23)	<0.001
TC (mmol/L)	4.83 (4.04, 5.68)	4.97 (4.10, 5.92)	4.78 (4.02, 5.60)	0.001
HDL-c (mmol/L)	0.99 (0.84, 1.16)	0.98 (0.84, 1.14)	0.99 (0.84, 1.17)	0.229
LDL-c (mmol/L)	2.71 ± 0.90	2.69 ± 0.94	2.71 ± 0.89	0.550
Cr (μmol/L)	62.0 (52.0, 73.0)	65.0 (51.0, 83.0)	62.0 (52.0, 71.0)	<0.001
eGFR (ml/min per 1.73 m^2^)	104.16 ± 18.44	95.91 ± 25.16	107.18 ± 14.13	<0.001
UACR (mg/g)	11.10 (5.90, 33.00)	107.10 (48.48, 337.50)	7.80 (5.00, 13.00)	<0.001
UA (μmol/L)	316.0 (261.0, 379.0)	329.0 (266.0, 401.5)	312.0 (260.0, 372.0)	<0.001
Antidiabetic agents currently(n, %)	1775, 67.98	543, 77.57	1232, 64.47	<0.001
Antihypertensive agents currently (n, %)	747, 28.61	312, 44.51	435, 22.77	<0.001

The data were displayed as means ± standard deviation or as median (interquartile range) for continuous variables, or numbers and percentage for categorical variables. DKD diabetic kidney disease, SBP systolic blood pressure, DBP diastolic blood pressure, BMI body mass index, WC waist circumference, NC neck circumference, NHR neck-to-height ratio, FBG fasting plasma glucose, HbA1c glycosylated hemoglobin A1c, TG triglyceride, TC total cholesterol, HDL-c high-density lipoprotein cholesterol, LDL-c low-density lipoprotein cholesterol, Cr serum creatinine, UACR urinary albumin-to-creatinine ratio, UA uric acid

### 3.2 Associations of NC and NHR with prevalent DKD

As shown in **Table 2**, after full adjustments for age, sex, diabetes duration, smoking and drinking status, SBP, TC, FBG, BMI, WC, antidiabetic agents currently and antihypertensive agents currently, the highest tertile of NC was not associated with prevalent DKD compared to the lowest tertile of NC (OR = 1.24, 95% CI: 0.87, 1.76). However, patients at the highest tertile of NHR were 1.63 times more likely to have DKD (OR = 1.63, 95% CI: 1.22, 2.18) than those at the lowest tertile of NHR in the same full-adjusted model.

**Table 2:** Associations of NC and NHR with the prevalence of DKD in patients with T2DM.

	Case/Participants (%)	Model 1			Model 2			Model 3		
		OR	95% CI	P Value	OR	95% CI	P Value	OR	95% CI	P Value
NC										
T_1_	174/687 (25.33)	1.00			1.00			1.00		
T_2_	227/866 (26.21)	1.45	1.13-1.88	0.004	1.29	0.98-1.69	0.071	1.17	0.88-1.56	0.290
T_3_	301/1062 (28.34)	1.98	1.50-2.61	<0.001	1.48	1.10-1.99	0.010	1.24	0.87-1.76	0.232
NHR										
T_1_	173/877 (19.73)	1.00			1.00			1.00		
T_2_	242/864 (28.01)	1.70	1.35-2.13	<0.001	1.48	1.16-1.88	0.002	1.43	1.11-1.85	0.006
T_3_	287/874 (32.84)	2.21	1.76-2.78	<0.001	1.77	1.39-2.25	<0.001	1.63	1.22-2.18	0.001

Model 1: age, sex; Model 2: Model 1 + diabetes duration, smoking and drinking status, SBP, TC, FBG; Model 3: Model 2 + BMI, WC, antidiabetic agents currently, antihypertensive agents currently. NC neck circumference, NHR neck-to-height ratio, DKD diabetic kidney disease, T2DM type 2 diabetes mellitus, OR odds ratio, CI confidence interval, SBP systolic blood pressure, TC total cholesterol, FBG fasting plasma glucose, BMI body mass index, WC waist circumference

The secondary analyses were further performed to explore the associations of NHR with levels of eGFR and UACR. Compared with the lowest one, the highest tertile of NHR was significantly associated with lower eGFR level (β = -4.64, 95% CI: -6.55, -2.74) and higher LnUACR level (β = 0.27, 95% CI: 0.10, 0.45) after full adjustments (**Table 3**).

**Table 3:** Associations between NHR and eGFR/LnUACR level in patients with T2DM.

NHR	Case/Participants (%)	eGFR^a^			LnUACR^b^		
		β	95% CI	P Value	β	95% CI	P Value
T_1_	173/877 (19.73)	1.00			1.00		
T_2_	242/864 (28.01)	-2.19	-3.84- (-0.55)	0.009	0.07	-0.08-0.22	0.381
T_3_	287/874 (32.84)	-4.64	-6.55- (-2.74)	<0.001	0.27	0.10-0.45	0.002

^a^The model was adjusted for sex, diabetes duration, smoking and drinking status, SBP, TC, FBG, BMI, WC, antidiabetic agents currently and antihypertensive agents currently.

^b^The model was adjusted for age, sex, diabetes duration, smoking and drinking status, SBP, TC, FBG, BMI, WC, antidiabetic agents currently and antihypertensive agents currently. NHR neck-to-height ratio, eGFR the estimated glomerular filtration rate, LnUACR log-transformed urinary albumin-to-creatinine ratio, T2DM type 2 diabetes mellitus, CI confidence interval, SBP systolic blood pressure, TC total cholesterol, FBG fasting plasma glucose, BMI body mass index, WC waist circumference

### 3.3 Subgroup analysis

Interaction effects were analyzed in strata of sex, age, diabetes duration, overweight/general obesity, central obesity, hypertension, and dyslipidemia after total adjustments. As presented in **Table 4**, participants at the highest tertile of NHR remained at a higher risk of DKD than those at the lowest tertile among most of the strata analyzed, except in those with older age, shorter diabetes duration, central obesity, and in those without overweight/general obesity or dyslipidemia. No interactive effects were observed in any of these strata (all *P* for interaction > 0.05)

**Table 4:** Subgroup analyses on the association of NHR with the prevalence of DKD in T2DM patients.

Subgroup	Case/Subjects (%)	OR	95% CI	P Value	P for interaction
Total	702/2615 (26.85)	1.63	1.22-2.18	0.001	
Sex					0.241
Male	435/1786 (24.36)	1.58	1.08-2.31	0.019	
Female	267/829 (32.21)	1.62	1.02-2.58	0.041	
Age					0.557
< 65 years	598/2348 (25.47)	1.62	1.18-2.20	0.003	
≥ 65 years	104/267 (38.95)	1.93	0.86-4.37	0.112	
Diabetes duration (median)					0.473
< 68 months	257/1289 (19.94)	1.52	0.97-2.37	0.066	
≥ 68 months	439/1292 (33.98)	1.75	1.19-2.57	0.005	
Overweight/ general obesity					0.562
No	257/1149 (22.37)	1.56	0.95-2.56	0.082	
Yes	445/1466 (30.35)	1.58	1.05-2.38	0.027	
Central obesity					0.577
No	292/1239 (23.57)	2.40	1.54-3.75	<0.001	
Yes	409/1371 (29.83)	1.31	0.87-1.97	0.196	
Hypertension					0.414
No	193/1345 (14.35)	1.65	1.02-2.67	0.043	
Yes	509/1270 (40.08)	1.59	1.10-2.30	0.013	
Dyslipidemia					0.266
No	198/898 (22.05)	1.37	0.81-2.30	0.237	
Yes	504/1717 (29.35)	1.91	1.34-2.72	<0.001	

Above analyses were adjusted for age, sex, diabetes duration, smoking and drinking status, SBP, TC, FBG, BMI, WC, antidiabetic agents currently and antihypertensive agents currently. NHR neck-to-height ratio, DKD diabetic kidney disease, T2DM type 2 diabetes mellitus, OR odds ratio, CI confidence interval, SBP systolic blood pressure, TC total cholesterol, FBG fasting plasma glucose, BMI body mass index, WC waist circumference.

## 4 Discussion

The current study was the first population-based epidemiological study to investigate the associations of NC and NHR, as indicators of upper-body subcutaneous fat, with prevalent DKD in Chinese population with T2DM. Our major finding indicated that NHR, instead of NC, was positively associated with the presence of DKD in patients with T2DM, independent of cardiometabolic risk factors. The increase in NHR was also related to a decrease in eGFR and an increase in UACR levels. Such discoveries suggested that NHR might be a potential indicator for identifying patients at a higher risk of DKD.

Studies about the relationship between NC and kidney dysfunction were limited and inconsistent. A research based on the general Chinese adults found the negative association between NC and eGFR (16). Similarly, a Korean community-based study also showed that eGFR was decreased in subjects with higher NC (17). However, a study including 177 patients with high cardiometabolic risk indicated that larger NC was related to the higher eGFR level (15). The disagreements with these studies might due to different health conditions of populations enrolled and distinct influencing factors considered. A Chinese research hold by Wan et al. showed that NC was positively associated with prevalent DKD, but without further adjusted for WC (8). WC, a proxy for central obesity, was found to be closely related to microalbuminuria and renal damage in patients with diabetes (25–27). Thereby, in order to testify the independent effect of NC on DKD, the impact of WC should be considered. Of note, our study found that the connection between NC and DKD disappeared with adjustments for WC. Moreover, in Xue et al.’s study (16), the negative association of NC with eGFR no longer existed in subjects with diabetes, which suggested that the relationship between NC and eGFR might be concealed by the strongly harmful effect of hyperglycemia on renal function. These results indicated that NC might be unstable and inaccurate to reveal kidney damage in patients with diabetes.

Likewise, several other studies have showed that in contrast to NC, NHR was more closely related to metabolic disorders, such as arterial stiffness, liver stiffness, obstructive sleep apnea syndrome (OSAS) and metabolic syndrome (MetS) (12, 13, 28–30). A community-based study demonstrated that the increase in NHR, rather than NC was related to higher brachial-ankle pulse wave velocity (baPWV) (12). An Indian study revealed that NC and NHR were both great indicators for MetS, but as to cardiovascular risk prediction, NHR was more plausible (29). It’s conceivable that NHR is more reliable than NC to represent for upper-body subcutaneous fat, since it considers the effect of height on whole body fat distribution. However, no studies to date have been done to explore the relationship of NHR with kidney dysfunction in any population. Our study based on Chinese patients with T2DM discovered that NHR was positively associated with prevalent DKD after full adjustments. What’s more, none of the interaction effects of cardiometabolic risk factors, which were all found to be closely related to DKD (1, 7, 31, 32), on the association between NHR and DKD were observed in our study. It indicated that the influence of NHR on DKD was not interfered by these cardiometabolic risk factors and further revealed the relative independence and stability of NHR in its relationship with DKD. Additionally, in consideration of BMI and WC as indicators for generalized and central fat accumulations respectively, our findings might indirectly prove that upper-body subcutaneous fat accumulation, represented by NHR, was indeed the unique fat site, which could confer extra metabolic risks beyond generalized and central obesity (33, 34).

The mechanisms for the association between excessive upper-body subcutaneous fat and the increasing risk of DKD remained unclear. Firstly, upper-body subcutaneous fat releases the majority of free fatty acids (FFAs) (35, 36), which could lead to the endothelial dysfunction and motivate the production of reactive oxygen species (37, 38), thereby having pathogenic effects on kidney, especially on tubulointerstitium (39) and podocytes (40–42). Secondly, larger upper-body subcutaneous fat is closely related to the increasing risk of IR (10, 43). While insulin sensitivity of the glomerular podocytes is vital for normal renal function (44), and previous studies have discovered that IR did propel the development of DKD (45, 46). Thirdly, patients with larger upper-body subcutaneous fat are more likely to have OSAS (47), the latter would accelerate the progress of DKD and other diabetic microvascular complications *via* promoting oxidative and nitrosative stress (48–50). At last, Mangge et al. proposed that nuchal fat accumulation, by secreting inflammatory cytokines and adipokines (51), might accelerate cell turnover and mitochondrial activity, thus result in telomeres damage and shortening (52). Shorter pieces of telomeres lead to senescent cells and ultimately influence phenotypes and functions of organs (53). Previous studies discovered that patients with shorter telomere length developed increased microalbuminuria, reduced eGFR and impaired kidney function (54–56). Telomere shortening may be another cause for the renal damage due to excessive upper-body subcutaneous fat.

There were several limitations in our study. Firstly, it was a cross-sectional study, causality between NHR and DKD cannot be established. Secondly, our study participants were from a single center and the great majority of them were hospitalized for relative poor glycemic control, it’s generalizability should be verified by involving outpatients or community patients in the future. Thirdly, direct adipose tissue measurements, such as CT or MRI are required to verify the authenticity of NHR. Cohort studies with larger and multicentric samples should also be done.

## 5 Conclusion

The present study demonstrated that the higher levels of NHR was significantly associated with the higher presence of DKD in Chinese patients with T2DM, independent of cardiometabolic risk factors. NHR might be a potential indicator for screening renal dysfunction in patients with T2DM, which needs more prospective studies to be confirmed in the future.

## Data availability statement

The datasets are available from the corresponding author on reasonable request. Requests to access these datasets should be directed to X-JG, guxuejiang@wmu.edu.cn.

## Ethics statement

The study involving human participants were reviewed and approved by the Ethics Committee in Clinical Research of the First Affiliated Hospital of Wenzhou Medical University. The patients/participants provided their written informed consent to participate in this study.

## Author contributions

Z-YH has made substantial contributions to the draft of the manuscript, and analysis of data. Z-YH, XG, L-JD, Y-QL, JL, and L-YP have made acquisition of data. XH, X-XZ, L-JY, BY, X-JG, and X-LL have been involved in revising the manuscript. X-JG and X-LL have given final approval of the version to be published. All authors contributed to the article and approved the submitted version.
